# Editorial: Environment and Skin Cancer

**DOI:** 10.3389/fonc.2022.924225

**Published:** 2022-05-27

**Authors:** Nabiha Yusuf, Thomas Haarmann-Stemmann, Motoki Nakamura

**Affiliations:** ^1^ Department of Dermatology, University of Alabama at Birmingham, Birmingham, AL, United States; ^2^ Department of Dermatology, IUF – Leibniz-Research Institute for Environmental Medicine, Düsseldorf, Germany; ^3^ Department of Environmental and Geriatric Dermatology, Graduate School of Medical Sciences, Nagoya City University, Nagoya, Japan

**Keywords:** ultraviolet radiation, non-melanoma skin cancer, melanoma, exposome, merkel cell carcinoma

The incidence of melanoma and non-melanoma skin cancer (NMSC) has been steadily increasing over the past decades (1). With millions of people affected worldwide, skin cancer poses a global threat for the health of the general population and a tremendous economic burden to health care systems (2). The major risk factor for the development of the vast majority of skin cancers, including basal cell carcinoma (BCC), squamous cell carcinoma (SCC), and melanoma, is a repetitive exposure to ultraviolet (UV) radiation. Second to polyomavirus infection, UV exposure is also major risk factor for the development of Merkel cell carcinoma (MCC), a rare but highly aggressive type of skin cancer (3). Whereas UVB radiation is absorbed by the DNA resulting in the formation of mutagenic DNA photoproducts, UVA rays interact with endogenous photosensitizers to induce oxidative stress and oxidative damage of DNA, proteins, and lipids (4). DNA damage-dependent and -independent responses stimulate pro-inflammatory, anti-apoptotic and immunosuppressive effects thereby facilitating the accumulation of damaged cells which may give rise to skin cancer. Accordingly, parameters such as the depletion of the ozone layer, the demographic development, and recreational behavior (sun bathing, tanning beds) sign responsible for the increasing numbers of skin cancers. However, next to UV radiation, exposure to various environmental chemicals, including arsenic compounds and combustion-derived polycyclic aromatic hydrocarbons (PAH), and even to therapeutic agents, such as sulfonamide-based protein kinase inhibitors, may induce skin carcinogenesis, by either directly damaging the DNA, causing oxidative stress, or interacting with signal transduction networks and transcription factors (5).

The identification of risk factors across the skin exposome and their mutual interaction, the elucidation of pathomechanisms and the identification of biomarkers is key to the development of novel preventive and therapeutic strategies for skin cancer. In this Research Topic of *Frontiers in Oncology* the authors shed light on different levels and facets of skin cancer development and biology which hopefully stimulates the field to enforce and improve the fight against this devastating disease.

Starting in the stratosphere, Umar and Tasduq focus on the depletion of the ozone layer, its impact on the UV index and the resulting consequences for human health. The authors also address potential adverse health effects of skin photoprotection, i.e. a vitamin D deficiency and associated disorders. The authors also contributed to another work emphasizing that autophagy positively regulates skin homeostasis by enhancing DNA damage recognition. Specifically, exposure of human dermal fibroblasts to UVB radiation impaired the autophagy response in a time- and intensity-independent manner, which was reversed after treatment with pharmacological activators, thus protecting against UVB radiation-induced photodamage [Umar et al.].

The occurrence of NMSC with advanced age and UVB exposure is specifically linked to diminished insulin-like growth factor-1 (IGF-1) signaling from senescent dermal fibroblasts in geriatric skin. Frommeyer et al. found that wounding therapies, such as dermabrasion, microneedling, chemical peeling, and fractionated laser resurfacing, restore IGF-1/IGF-1R signaling in geriatric skin. Wondrak and co-workers report about another potential therapeutic approach involving hypochlorous acid (HOCl), an agent that has emerged as an important component of the skin exposome. While exploring the interaction between solar UV exposure and environmental HOCl exposure, they identified an unrecognized photo-chemopreventive activity of topical HOCl and associated chlorination stress that blocked tumorigenic inflammatory progression in UV-induced high-risk mouse skin [Snell et al.].

Nurzat et al. investigate the functional role of different integrin alpha/beta (ITGA/ITGB) subunits in cutaneous melanoma. Bioinformatic analysis tools were used to identify abnormally-expressed genes and gene regulatory networks associated with melanoma, which may improve our understanding of the melanoma pathogenesis. Specifically, they found that the expression level of various ITGA and ITGB subunits was associated with immune cell infiltration, metastasis, and disease-free and overall survival. Vogeley et al. focus on environmental and occupational risk factors, in particular UV radiation and PAHs, which initiate the development of cutaneous SCC, at least in part, by activating the aryl hydrocarbon receptor and impairing defense mechanisms, such as DNA repair, apoptosis and anti-tumor immune responses. Apart from environmental and occupational stressors, certain therapeutic drugs induce the development of cutaneous SCC as an off-target effect. The BRAF inhibitor vemurafenib, approved for treating patients with BRAF V600E-mutant melanomas, for instance, causes various cutaneous adverse events, including hyperkeratotic skin lesions and cutaneous SCCs. Tham et al. report that both cutaneous adverse events are under direct control of vemurafenib-dependent MEK-ERK hyperactivation and confirm the dependence on preexisting genetic alterations in epidermal keratinocytes that predispose to carcinogenesis.

Another protein involved in carcinogenesis, in particular in the pathogenesis of melanoma, is the plasminogen activating inhibitor-1 (PAI-1). Several reports indicate pro-tumorigenic functions of PAI-1 in cancer progression and metastasis, for instance controlling PD-L1 expression. Moreover, baseline serum levels of PAI-1 were significantly decreased in therapy responders compared to non-responders. These results suggest that baseline serum levels of PAI-1 may be useful as a biomarker for identifying patients who would respond to anti-melanoma immunotherapy [Ohuchi et al.]. Irrespective of molecular pathomechanisms, Chang et al. assess whether and to which extent comorbidities and stages may influence the prognosis of melanoma patients. In a retrospective cohort study by using the national health insurance research database in Taiwan, a higher risk of mortality was found in patients who had localized tumors, regional metastases, or distant metastases with more comorbidity scores.

Wijaya et al. conducted a systematic review and meta-analysis to assess the association between MCC polyoma virus (MCPyV) infection and MCC, non-MCC skin lesions, and healthy skin. MCPyV infection significantly increased the risk for MCC. However, the low prevalence of MCPyV in non-MCC skin lesions did not exclude a pathogenic association of this virus with the development of non-MCC skin lesions. Staying in the MCC context, Nakamura et al. investigated the prognostic value of tertiary lymphoid structures (TLSs) in patients suffering from MCPyV-positive and MCPyV-negative MCC. They found that TLSs can indeed serve as prognostic biomarker for MCC patients, even in cohorts encompassing MCPyV-negative, thus UV-induced cases. Furthermore, the assessment of TLS-associated chemokine profiles may enable a better understanding of the tumor microenvironment in patients with MCPyV-positive or MCPyV-negative MCC.

## Author Contributions

NY wrote the draft. TH and MN edited the draft and finalized the current version. All the authors made substantial intellectual contribution and approved the article for publication.

## Funding

This work was supported by 1R01AR071157-01A1 (Nabiha Yusuf) from the National Institute of Arthritis and Musculoskeletal and Skin Diseases.

## Conflict of Interest

The authors declare that the research was conducted in the absence of any commercial or financial relationships that could be construed as a potential conflict of interest.

## Publisher’s Note

All claims expressed in this article are solely those of the authors and do not necessarily represent those of their affiliated organizations, or those of the publisher, the editors and the reviewers. Any product that may be evaluated in this article, or claim that may be made by its manufacturer, is not guaranteed or endorsed by the publisher.
